# Sequence Features of Mitochondrial Transporter Protein Families

**DOI:** 10.3390/biom10121611

**Published:** 2020-11-28

**Authors:** Gergely Gyimesi, Matthias A. Hediger

**Affiliations:** Membrane Transport Discovery Lab, Department of Nephrology and Hypertension, and Department of Biomedical Research, Inselspital, University of Bern, Kinderklinik, Freiburgstrasse 15, CH-3010 Bern, Switzerland; matthias.hediger@ibmm.unibe.ch

**Keywords:** mitochondrial carriers, SLC transporters, SLC25, MCF, SLC54, MPC, SLC55, LETM, SLC56, sideroflexin, ABC transporter, sequence analysis, protein targeting

## Abstract

Mitochondrial carriers facilitate the transfer of small molecules across the inner mitochondrial membrane (IMM) to support mitochondrial function and core cellular processes. In addition to the classical SLC25 (solute carrier family 25) mitochondrial carriers, the past decade has led to the discovery of additional protein families with numerous members that exhibit IMM localization and transporter-like properties. These include mitochondrial pyruvate carriers, sideroflexins, and mitochondrial cation/H^+^ exchangers. These transport proteins were linked to vital physiological functions and disease. Their structures and transport mechanisms are, however, still largely unknown and understudied. Protein sequence analysis per se can often pinpoint hotspots that are of functional or structural importance. In this review, we summarize current knowledge about the sequence features of mitochondrial transporters with a special focus on the newly included SLC54, SLC55 and SLC56 families of the SLC solute carrier superfamily. Taking a step further, we combine sequence conservation analysis with transmembrane segment and secondary structure prediction methods to extract residue positions and sequence motifs that likely play a role in substrate binding, binding site gating or structural stability. We hope that our review will help guide future experimental efforts by the scientific community to unravel the transport mechanisms and structures of these novel mitochondrial carriers.

## 1. Introduction

Mitochondria are believed to have evolved through an endosymbiotic event, where an α-proteobacteria has been engulfed by a host cell, possibly an archaeon [1,2]. This event is thought to have arisen only once, and in the last 2 billion years, mitochondria have evolved together and in close concordance with their host cells [1,3,4]. During this time, significant changes in the genome of the endosymbiont have taken place, which involved the transfer of most mitochondrial proteins to the nucleus, as well as the emergence of novel protein families in the nuclear genome that are targeted to mitochondria [4,5]. The vestigial mitochondrial genomes of vertebrates in general code for 13 internal membrane proteins that are involved in electron transport and coupled oxidative phosphorylation [6,7], while the part of the mitochondrial proteome related to transmembrane transport, i.e., the exchange of metabolites and ions with the host cell, is almost exclusively of eukaryotic origin [4,5].

Proteins in the human inner mitochondrial membrane (IMM) are quite distinct from one another in terms of their structure and sequence features as well as their trafficking and import mechanisms into mitochondria. Based on this diversity, they are likely polyphyletic in origin and presumably arose independently of each other. Membrane proteins that take part in transmembrane solute transport across the IMM include members of the following families: SLC25 (mitochondrial carriers), SLC8 (SLC8B1/NCLX Na^+^/Ca^2+^/Li^+^ exchanger), SLC54 (MPC, mitochondrial pyruvate carriers), SLC55 (LETM, leucine zipper-EF-hand-containing transmembrane proteins), SLC56 (sideroflexins), ATP-binding cassette (ABC) transporters (ABCB7, ABCB8, ABCB10) and various ion channels [8]. The discovery, biological function, physiological role and disease involvement of many of these transport proteins and channels are comprehensively presented in excellent articles of the present review series. In particular, ion channels are covered in detail in the review by Szabo et al. and will not be discussed here. Metabolite and ion transport between the cytoplasm and the mitochondria also require the broad-specificity channels of the outer mitochondrial membrane (OMM). The most prominent class of such channels, the voltage-dependent anion channels (VDACs), is discussed in detail by Shoshan-Barmatz et al. in this review series [9], but other OMM transporters with unknown substrate specificity may also be present [10]. In this review, we focus on the discussion of specific structure and sequence features that shape trafficking and functional properties of transporter-like proteins in the IMM.

### Trafficking of Membrane Transporters into Mitochondria

**Figure 1:**
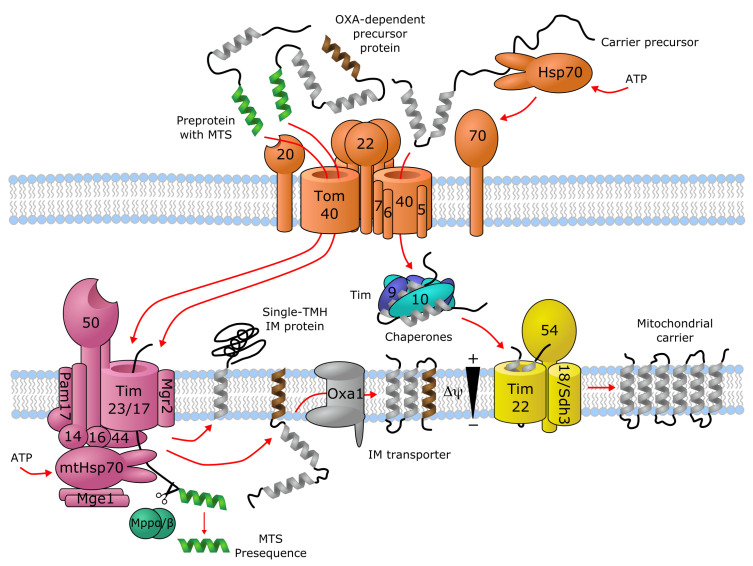
Import machinery for mitochondrial transporters. The TOM (orange), TIM23 (purple) and TIM22 (yellow) complexes are depicted, with numbers representing the corresponding Tom/Tim protein names. Proteins with a single transmembrane helix (TMH) and OXA-dependent transporters employ the TIM23 import pathway, while carriers with no mitochondrial targeting sequence (MTS) are imported through TIM22. For details, see text. Figure is based on [11,12,13,17,18,19].

Transporters of the mitochondrial inner membrane are synthesized in the cytoplasm and imported into the mitochondria through specialized import machinery [11]. In contrast to most mitochondrial proteins, most mitochondrial carriers typically do not contain an N-terminal mitochondrial targeting sequence (MTS). Instead, the nascent precursor transporter proteins bind to the ATP-hydrolyzing Hsp70 and Hsp90 chaperones in the cytoplasm, which deliver them to the translocase of the outer membrane (TOM) complex. Here, the Tom70 receptor, part of TOM, binds both the precursor protein and the chaperones, and transfers the precursor protein to Tom22, where it then gets translocated in a loop-wise fashion through Tom40, the channel component of TOM [12,13]. Once in the intermembrane space, the hydrophobic regions of the precursor transporter proteins are shielded by the heterohexameric chaperone complex Tim9-Tim10-Tim12. This complex of the precursor protein and the chaperones then binds to the receptor-like protein Tim54, which is part of the translocase of the inner membrane (TIM22) complex in the IMM. Here, another member of TIM22, the channel-forming Tim22 protein, then inserts the precursor protein into the inner mitochondrial membrane [11] (Figure 1).

As an alternative import mechanism, certain precursor transporter proteins do carry the MTS on their N-termini, which typically forms a short (15–50 residues) amphipathic helix with a net positive charge [14,15]. Transporters containing an N-terminal MTS, such as SLC55/LETM and mitochondrial ABCB transporters, are delivered from the cytoplasm by the Tom20 receptor of the TOM complex, recognizing the hydrophobic side of the amphipathic helix formed by the MTS [16]. Upon transfer to the intermembrane space through Tom40, the targeting sequence binds to the Tim50 receptor component of another inner membrane complex, TIM23 [17]. This activates the Tim23 channel subunit of the TIM23 complex to allow the translocation of the bound precursor protein through the IMM [11]. Precursor proteins that are destined to the lipid bilayer contain a hydrophobic stop-transfer signal sequence, which is recognized within the IMM by the small transmembrane protein Mgr2, initiating the lateral release of the imported precursor protein into the membrane [11,18,19] (Figure 1). Interestingly, an alternative mechanism exists, where Oxa1, the main component of the oxidase assembly (OXA) translocase complex, inserts the hydrophobic segment into the inner membrane bilayer after they pass through the Tim23 pore [11] (Figure 1). It has been shown that even a single protein with multiple membrane-spanning segments can use different mechanisms to import individual transmembrane segments into the membrane, such as the yeast protein Mdl1, a mitochondrial ABCB family homolog [20]. Further details about the molecular machinery for importing metabolite transporters into mitochondria are discussed by Rampelt et al. as part of the present special review series.

Hydrogenosomes and mitosomes are cellular organelles that share a common evolutionary origin with mitochondria [21], and the comparison of their protein import machineries has shed light on important events shaping the early evolution of the mitochondrial import machinery [22]. Protein precursors destined to the hydrogenosomes and mitosomes may contain N-terminal presequences that are typically shorter than MTSs and also lack a marked positive charge [22]. Nevertheless, various hydrogenosomal proteins from the primitive eukaryote *Trichomonas vaginalis* readily target into mitochondria when expressed in yeast, and vice versa [22]. Furthermore, the Tom20 receptor and the acidic N-terminal extension of the Tom22 receptor, both playing fundamental roles in the recognition of the positively charged amphipathic presequence, seem to have evolved independently after the last common eukaryotic ancestor [23], indicating that the primitive TOM import complex did not have these features. All these arguments suggest that the MTS and the corresponding recognition and import machinery evolved in a convergent way in various eukaryotic lineages on top of a pre-existing ancestral protein import machinery, which was most likely independent of the MTS [22]. Based on this reasoning, proteins utilizing the conserved MTS-independent import pathway, such as most SLC25 carriers, might have been one of the earliest groups of proteins that developed mitochondrial localization.

The development of a positively charged MTS has been linked to the appearance of the electron transport chain, which resulted in a markedly negative membrane potential across the IMM [22]. In this scenario, a positively charged presequence would confer an evolutionary advantage during the membrane translocation of the precursor protein due to the electrophoretic effect [22]. In addition, a functional MTS can arise de novo fairly easily through random mutations or DNA rearrangements [24,25,26,27]. The development of an MTS-dependent import machinery with an easily generatable MTS could thus have promoted the evolvability of the host organism [27].

## 2. SLC25—Mitochondrial Carrier Family (MCF)

The largest protein family of mitochondrial solute transporters is the SLC25 (mitochondrial carrier) family. In human there are a total of 53 members that fulfil the vital roles of uniport or exchange of ions, metabolites and other solutes across the IMM [28]. It has been recognized early on that the sequence of the ADP/ATP translocase (SLC25A4) has a repeating sequence element that contains two hydrophobic segments and repeats 3 times in the sequence [29]. Such an internal repeat symmetry is commonly found in membrane transporters [30]. Indeed, such features were later found in several other members of the protein family [31,32,33,34], along with conserved proline, glycine and acidic amino acid residues [35]. The conserved residues were later compiled into a consensus characteristic or “signature motif” for mitochondrial carriers, Px(D/E)xx(K/R) [36], and the conserved charged residues were indeed found to take part in specific conserved salt-bridge contacts, termed the matrix network [37,38,39,40,41,42], in the 3D structure of the proteins [43,44]. The signature motif can be extended in the C-terminal direction to include a proximal glutamine (Q) residue, which helps stabilize the salt-bridge contacts on the matrix side of the carrier, forming the “Q brace” [44,45]. There is a similar cluster of charged residues on the intermembrane side of the even-numbered TM helices, forming the cytoplasmic salt-bridge network, where the charged residues of a (Y/F)(D/E)xx(K/R) motif engage in salt-bridge contacts [42,44]. This network is stabilized by the so-called tyrosine (Y) brace, formed by the hydrogen bonding of the tyrosine residues of the motif to these salt bridges [45]. A further sequence motif, (Y/W/L/F)(K/R)GxxP, present in the connecting loop between each short matrix helix and the following transmembrane helix, has been described, replacement of which disrupts the function of the transporter [46]. In addition, close helix-helix contacts in the matrix-facing state are formed by two conserved sequence motifs, πGπxπG on the odd-numbered, and πxxxπ on the even-numbered helices, where π stands for a residue with a small side-chain [45,47,48]. Further details of these sequence motifs, including their roles in the transport mechanism and a functional interpretation of individual amino acid residues is covered in detail by Kunji et al. in this special issue. In particular, disease-causing mutations in context of the 3D structure of SLC25 carriers and their sequence motifs are extensively reviewed by Palmieri et al. in the present review series [49].

Interestingly, the sequence motif (QYKGxxDCxRK) in the short matrix helices has also been described, which is only conserved in a subset of mitochondrial carriers, such as ADP/ATP (SLC25A4–6, SLC25A31), aspartate/glutamate (SLC25A12–13), ornithine (SLC25A2, SLC25A15), glutamate (SLC25A18, SLC25A22), and carnitine (SLC25A20) carriers, one ATP/P_i_ carrier (SLC25A24) and three carriers with unknown function (SLC25A9, SLC25A34, SLC25A45) [50]. This motif was proposed to harbor residues that go through post-translational modification thereby locally altering the protein structure and thus modulating function. One example for this is acetylation at K163 of SLC25A5 (AAC2), corresponding to the last residue of the motif, while the cysteine residue of the motif might interact with oxidizing/reducing agents [50]. Further investigation is required to reveal the precise functional role of this sequence motif.

Most SLC25 family proteins do not contain an MTS at their N-termini. Instead, they seem to hold mitochondrial targeting information in all three segments of their three-fold repeat sequence [51,52]. SLC25 proteins are embedded into the IMM by the TIM22 machinery, as described in the previous chapter. It was suggested that the three repeating segments of SLC25 proteins act in a cooperative manner to facilitate receptor binding and translocation into mitochondria [53], nevertheless, a single unit consisting of a matrix loop and the following transmembrane helix is enough for mitochondrial localization [52]. The net positive charge of the short matrix loop helices have been shown to be essential for import into mitochondria, and these regions are thought to interact with the Tom40 channel component of the TOM complex, which effectively functions as a selectivity filter [52].

Interestingly, certain SLC25 proteins, such as the mitochondrial phosphate carrier (SLC25A3) and citrate/tricarboxylate carrier (SLC25A1) have been proposed to harbor an N-terminal MTS, based on physicochemical composition of their N-terminal sequences and observation of mature protein forms truncated at the anticipated cleavage site [31,54]. This is also partially supported by TargetP-2.0 predictions, which report an MTS of 49 amino acids with likelihood 0.5749 for SLC25A3, exactly as anticipated from experiments where a protein fragment N-terminally truncated at the same position was identified [31]. For SLC25A1, the presence of an MTS was predicted with likelihood 0.3753. Whether a functional N-terminal MTS is present in these proteins has not been investigated experimentally. For all other human SLC25 proteins, the likelihood of an MTS at the N-terminus was less than 0.17 according to TargetP-2.0 predictions.

## 3. SLC54—Mitochondrial Pyruvate Carriers (MPC)

The mitochondrial pyruvate carriers have been identified as IMM transporters responsible for pyruvate uptake into the mitochondria [55,56]. MPC1 (SLC54A1) and MPC2 (SLC54A2) function as heterodimers [56,57], while humans as well as other placental mammals also contain a paralog of MPC1 called MPC1L (SLC54A3), the two sharing 48.2% sequence identity in human [58]. A detailed discussion of the biological role and function of MPCs and their links to disease can be found in a comprehensive review by Martinou et al. and Taylor et al. in this special issue.

MPCs have been predicted to harbor 2–3 transmembrane helices (TMHs) [55,56,59]. Interestingly, they are unrelated to SLC25 carriers and instead have been shown to be homologous to the 3-TMH repeating element in SWEET (“Sugars will eventually be exported transporters”) transporters [60,61], which show a 3 + 1 + 3 TMH architecture [62,63]. Proteins from the SWEET family also exist as homodimers of half-transporters encompassing the 3-TMH repeat, called SemiSWEET [62,64]. While there are relatively few studies on the structure and transport mechanism of MPCs, the structure and mechanism of SWEET and SemiSWEET transporters are quite well described [62,65,66,67,68,69]. Based on the suggested similarity, it can be speculated that the structure of a functional MPC transporter is similar to those of the homodimers of SemiSWEET proteins, or a single protomer of a SWEET-fold transporter. Based on this proposed similarity and the available structures for SWEET and SemiSWEET transporters, it should be possible to interpret disease-causing mutations in a structural context in the future, such as L79H and R97W in human SLC54A1/MPC1 [55,70,71].

**Figure 2:**
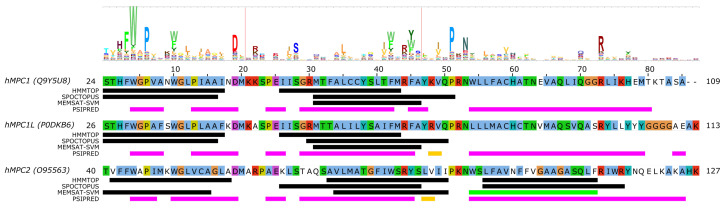
Sequence analysis of human MPC (SLC54) proteins. The diagram above the alignment shows the part of the HMM logo corresponding to the sequence (see text). At each position in the HMM logo, higher columns correspond to higher sequence conservation, and letters are drawn proportional to their frequency of occurrence at that position. Vertical lines in the HMM logo show positions with non-zero insertion frequency. Transmembrane regions predicted by three different methods (HMMTOP, SPOCTOPUS, MEMSAT-SVM) are marked by black lines. Green lines mark transmembrane regions predicted to by pore-lining by MEMSAT-SVM. Secondary structure as predicted by PSIPRED is shown as magenta lines (α-helix) and golden lines (β-strand). Uniprot sequence identifiers are shown next to protein names for each sequence.

Nevertheless, even without a structural context, one can analyze residue conservation in MPC proteins based on sequence alignment and the help of the “MPC” (PF03650) domain from the Pfam database [72]. The information of residue conservation encoded by profile hidden Markov models (HMM) of Pfam domains can be visualized as a HMM logo by a suitable software, such as Skylign [73] (Figure 2). We submitted the amino acid sequences of human SLC54A1–3 to three different methods to predict the location of transmembrane regions (HMMTOP [74], SPOCTOPUS [75], MEMSAT-SVM [76]) and secondary structural elements (PSIPRED [77]). Combining information on conservation with transmembrane region prediction pinpoints possible conserved charged/polar residues in transmembrane regions, which would imply that they might have a functional or structural role. Such residues may be S52/S54/S68, R68/R70/R84, H84/H86/N100 in MPC1/MPC1L/MPC2, respectively (Figure 2). Interestingly, residues N33/S35/K49 in MPC1/MPC1L/MPC2, respectively, represent a position which shows slight preference for polar/charged residues, but is asymmetric between MPC1/SLC54A1 and MPC2/SLC54A2 proteins, which could hint at a possible substrate-binding role [42]. We can also observe based on Figure 2 that the disease-associated mutation R97W [55] modifies an amino acid at a location that is considerably conserved in the family with a preference for basic sidechains, explaining the deleterious effects of the mutation. Intriguingly, the other currently known point mutation, L79H, is located at a poorly conserved position, and so is not expected to have a direct impact on the structure or function of the mature protein monomer. Several positions might contain structurally important residues with a clear preference for aromatic sidechains, such as F27/F29/F43, W28/W30/W44, W34/W36/W50, F66/F68/W82, F69/F71/Y85 in MPC1/MPC1L/MPC2, respectively. The detailed investigation of these residues in future studies might reveal more about their role in transporter function.

## 4. SLC56—Sideroflexins

Sideroflexin 1 (SLC56A1/SFXN1) has been described as the gene whose defects are responsible for the flexed-tail mouse phenotype with sideroblastic anemia [78]. Later, it was found that SLC56A1/SFXN1 plays a role in serine transport into the mitochondria, which in turn fuels one-carbon metabolism [79]. SLC56A3/SFXN3 and SLC56A2/SFXN2 can compensate for this function, as well as yeast and Drosophila homologues, indicating an ancestral function, while SLC56A4/SFXN4 and SLC56A5/SFXN5 were unable to do so, indicating a possible altered transport rate or substrate selectivity [79]. Regarding their mitochondrial targeting, sideroflexins do not show any canonical MTS [78], and instead are targeted to the mitochondria via the TIM22 import complex [80,81,82], similarly to SLC25 proteins.

**Figure 3:**
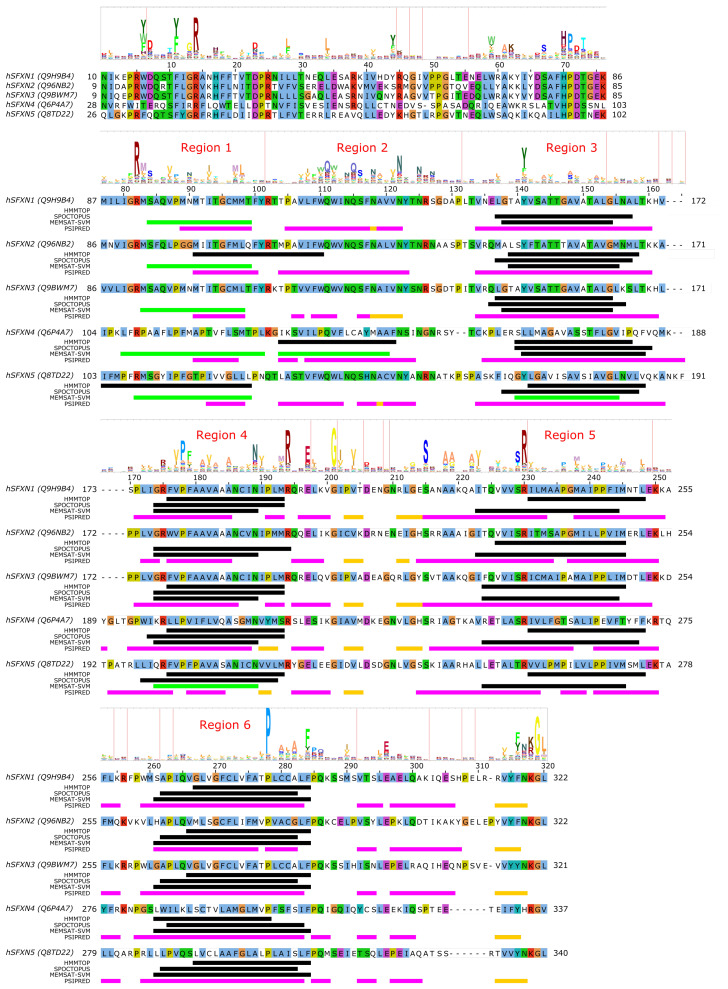
Sequence analysis of human sideroflexin proteins (SLC56 family). The HMM logo is shown above the sequence alignment (see text). Vertical lines in the HMM logo show positions with non-zero insertion frequency. Transmembrane regions predicted by three different methods (HMMTOP, SPOCTOPUS, MEMSAT-SVM) are marked by black lines. Green lines mark transmembrane regions predicted to by pore-lining by MEMSAT-SVM. Secondary structure as predicted by PSIPRED is shown as magenta lines (α-helix) and golden lines (β-strand). Uniprot sequence identifiers are shown next to protein names for each sequence.

Very little is currently known about the structure and function of sideroflexin transporters. No structural homologs exist and, interestingly, no internal repeat symmetry has been described for SLC56 proteins. Five transmembrane helices were proposed to be conserved among family members and a few consensus motifs have been described [78]. Nevertheless, sequence analysis can potentially hint at functionally important regions in SLC56 proteins. Since the 3D structure and thus the membrane-spanning regions of SLC56 proteins are not known, we submitted the amino acid sequences of human SLC56A1–5 to various methods to predict the location of transmembrane regions and secondary structural elements as for MPC/SLC54 proteins. Interestingly, while four regions are more-less robustly predicted as transmembrane by various methods (Figure 3, alignment positions 140–160, 175–195, 225–250, 260–285, marked as Region 3–6, respectively), there appears to be significant inconsistency in predicting the first TM helical region (alignment region 75–125). The predictions by HMMTOP suggest two distinct regions (alignment positions 75–100 and 105–125, marked Region 1 and 2), and a third region overlapping with the first two (alignment position 90–110). However, the third region seems less likely due to the lack of sequence conservation as reported by the HMM logo, and the probability of residue insertions in alignment region 100–105 (Figure 3). SPOCTOPUS consistently does not report a TM segment in alignment region 75–125, while MEMSAT-SVM consistently reports the presence of a TM segment in Region 1 (Figure 3). Interestingly, MEMSAT-SVM also reports a sixth TM segment in Region 2 in SLC56A4, which is also predicted to be transmembrane by HMMTOP. This region is predicted to be helical by PSIPRED, and marked as pore-lining by SPOCTOPUS, likely hinting at its amphipathic nature. Region 2 also contains a sequence motif that is seemingly conserved in the protein family, and that has been described as “asparagine rich” already after the identification of SLC56A1/SFXN1 [78]. This motif could be described by the consensus WQWxNQSxNxxxN motif, where polar residues N, S and Q are in conserved positions. According to PSIPRED predictions, occasional coil and strand content can occur near the second Q and second N residues (Figure 3). Such local distortions of helical geometry typically signal the location of substrate-binding sites in transmembrane transporter proteins [42,43,83,84], which, combined with the conservation pattern of residues, suggests that this region could have a functional role. Given that this putative functional region is often missed by transmembrane segment prediction software, while region 75–100 is consistently predicted by MEMSAT-SVM as transmembrane, combined with the presence of a non-conserved, insertion-prone region at 100–105, it is tempting to speculate that in fact regions 75–100 and 105–125 constitute two independent transmembrane helices, leading to an overall topology with six transmembrane helices in all SLC56 proteins.

Analysis of the other regions proposed as transmembrane also show remarkably conserved polar or charged residues that might play a role in shaping transport function. In Region 1, a conserved Arg residue is apparent from the HMM logo (Figure 3), corresponding to R92/R91/R91/R109/R108 in SLC56A1–5, respectively. Region 3 contains a conserved aromatic/hydrophobic residue (Y151/Y150/Y150/L166/Y167 in SLC56A1–5, respectively). Region 4 shows the consensus sequence pattern RxVPFxxVxxAxxxNxxxMR, of which P181/P180/P180/P201/P204 (SLC56A1–5, resp.) are conserved, indicating a possible structural role, while the N192/N191/N191/N212/N215 residues in the second half of the proposed transmembrane helix might play a functional role, and the highly conserved basic residue R197/R196/R196/R217/R220, proposed to be at the edge of the TM helix, might play a role in gating. Region 5 contains a conserved Ser-Arg (SR) motif with R233/R232/R232/R253/R256 highly preferred to be basic. In Region 6, the sequence pattern PxAxAxFPQ is apparent, with the first proline residue (P281/P280/P280/P301/P304, SLC56A1–5, resp.) being highly conserved, suggesting the presence of a functionally or structurally relevant kink in the transmembrane helix in this region. Interestingly, regions outside the predicted transmembrane segments also contain seemingly conserved sequence motifs. The region just N-terminal to the first predicted transmembrane segment (Region 1) harbors the HPDT motif that is well conserved in human sideroflexin proteins and has been recognized early on [78]. The proposed loop between Regions 4 and 5 also contains some remarkably conserved amino acid residues, such as E200/E199/E199/E220/E223, S218/S217/S217/S238/S241, (in SLC56A1–5, resp.), which might play a functional role; and G204/G203/G203/G224/G227, which could be important in maintaining structure, such as close helix-helix contacts. Finally, the last five C-terminal residues of human sideroflexin proteins contain the consensus sequence FNKGL of unknown function, with a highly conserved glycine residue. Notably, no disease-linked genetic defects that modify a single amino acid position have been described for SLC56 proteins so far, instead, only mutations causing frameshift and/or premature termination have been reported [85].

Since the above descriptions are only based on computer predictions, they are speculative at this point. However, given the current scarcity of structure-function studies on sideroflexin proteins, these amino acid positions can be helpful to identify functionally relevant hot spots. These hypotheses, however, await validation by future experimental studies.

## 5. The SLC55/LETM Mitochondrial Cation/Proton Exchanger Family

**Figure 4:**
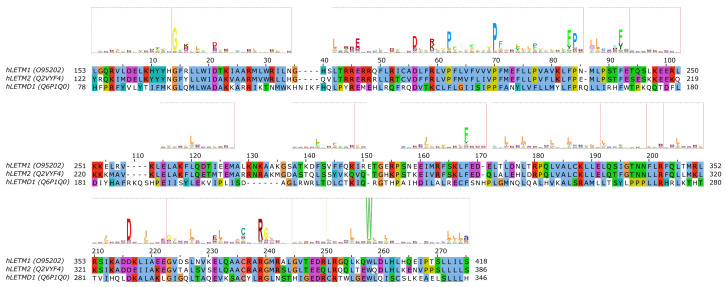
Sequence analysis of human LETM proteins (SLC55 family). HMM logo is shown over the alignment, based on the “LETM1” HMM model from Pfam (see text). Only the alignment region covered by the HMM model is shown. Certain non-conserved residue positions with ambiguous matches with the protein sequences have been removed from the HMM logo.

LETM1 (SLC55A1) has had a controversial role, as it was first proposed to be a part of the mitochondrial K^+^/H^+^ exchanger (KHE) pathway [86,87]. However, later, through a genome-wide genetic screen, it was found to be responsible for altered Ca^2+^ levels in mitochondria [88], and subsequently the purified LETM1 protein in liposomes was shown to mediate Ca^2+^/H^+^ exchange [88,89,90]. However, later it was argued that K^+^/H^+^ and unspecific and electroneutral cation/H^+^ exchange better explain the effect seen upon knockout of LETM1 in model cells and the effects seen in the mitochondria of model animals and patients with LETM1 deletion [91]. For a more detailed review of mitochondrial K^+^ homeostasis, see the comprehensive reviews by Szabo et al. and Szewczyk et al. in this special issue. Nevertheless, the structure of LETM1 was proposed to be a hexamer and the predicted single TMH (alignment region 60–80 in Figure 4 [86]) contains a conserved acidic residue (E221), the mutation of which causes loss of the ability for the protein to take up Ca^2+^ into liposomes [90], which can be interpreted as E221 being part of the substrate-binding site. Interestingly, this residue is not conserved in LETMD1/SLC55A3 (N151, Figure 4), while other polar residues are present in the transmembrane region, such as S145, hinting at a substrate spectrum or a function that is likely different from LETM1/SLC55A1 and LETM2/SLC55A2. Nevertheless, the transport mechanism of the protein remains unknown, even though it has been shown that a change in external pH causes conformational changes in LETM1 [90]. In the proposed transmembrane region of SLC55 proteins, two proline residues seem to be conserved based on our sequence alignment and the HMM logo (Figure 4, P211/P180/F140 and P219/P188/P148 in SLC55A1–3, respectively). Conserved proline residues often signal positions with structurally important kinks in helices [92,93]. LETM1 contains the mitochondrial signal peptide [90], similarly to LETM2 (likelihood 0.3338) and LETMD1 (likelihood 0.9889) according to TargetP-2.0 [94] predictions, indicating that SLC55 proteins likely get translocated into the mitochondria via the TIM23 translocation complex. Currently, no single-point mutations linked to disease have been reported for SLC55 proteins.

## 6. ATP-Binding Cassette (ABC) Transporters in Mitochondria

Currently, 3 of the 48 human ATP-binding cassette (ABC) transporters have been shown to be present in mitochondria, which are ABCB7, ABCB8 and ABCB10 (also known as ABC7 [95], M-ABC1 [96], and M-ABC2 [97], respectively). ABCB6 has also been suggested to be a mitochondrial transporter [98], but this has later been challenged [99,100]. The three mitochondrial ABC transporter proteins, in contrast to solute carriers, contain a mitochondrial targeting sequence at their N-termini [95,96,97,101], indicating that they are most likely translocated into the mitochondrial inner membrane via the TIM23 machinery. The presence of an N-terminal cleavable targeting signal is also supported by TargetP-2.0 predictions (likelihoods 0.9634, 0.7184, and 0.207 for human ABCB7, ABCB8, and ABCB10, respectively), while human ABCB6 was not predicted to contain a signal peptide (likelihood 0.0001).

A yeast ABC transporter protein (Mdl1) showing considerable sequence similarity to human ABCB proteins [102], was shown to use both the conservative Oxa1-mediated import machinery and lateral release from the TIM23 complex to translocate to the IMM [20]. Based on this, it is likely that human ABCB proteins also use the same pathway for mitochondrial targeting. In addition, the homologous yeast Mdl2 protein and Atm1 (human ABCB7 ortholog) have also been suggested to employ OXA-mediated membrane insertion into the IMM [103]. Nevertheless, while it is plausible that human mitochondrial ABCB proteins also employ a similar mechanism, this has not yet been experimentally studied.

ABCB transporters in the mitochondria are so-called “half transporters”, consisting of one nucleotide-binding domain (NBD) and one transmembrane domain (TMD), with homodimeric complexes forming the functional transporter unit. Recent structural studies of human ABCB10 [104] and yeast Atm1 [105] have highlighted conserved sequence motifs that are located along cavities forming the putative substrate-binding site in the TMD. Interestingly, they are not lying in overlapping locations in the two different proteins. For human ABCB7, residues R315, R319, N378, N425, T429, R432 and E433 (corresponding to R280, R284, N343, N390, S394, R397, D398 in yeast Atm1, respectively) in TMH4, TMH5 and TMH6 near the cytoplasmic membrane interface have been proposed to take part in substrate binding based on the yeast Atm1 structure with bound glutathione [105]. While one of these positions, E433 have been found to be mutated to lysine in patients with X-linked sideroblastic anemia (XLSA) [106], biochemical validation of these binding site residues is still missing [105]. For human ABCB10, a conserved signature sequence of (N/I)xxR (containing N229 and R232) and NxxDGxR (containing N289, D292, R295) in TMH2 and TMH3, respectively, were found and have been proposed to take part in substrate binding [104]. In the case of ABCB10, the missing identity of the transported substrate hampers biochemical studies on the functional role of individual residues. It has been proposed that ABCB10 transports an intermediate in the heme biosynthesis pathway [107]. Structure-function studies elucidating functionally relevant residues or sequence motifs of human ABCB8 are still missing. In neither ABCB8 nor in ABCB10 have disease-linked single-point mutations been described yet.

## 7. Mitochondrial Calcium Transport via SLC8 Family

SLC8B1 (NCLX, Na^+^/Ca^2+^/Li^+^ exchanger) was identified as the ion exchanger protein responsible for the exit of Ca^2+^ from the mitochondria [108]. SLC8B1 is thought to exchange 3 Na^+^ ions for 1 Ca^2+^ ion based on similarity to other Na^+^/Ca^2+^ exchangers [109,110,111], and is unique in the property that Li^+^ ions can replace Na^+^ ions in transport [112]. Recently, it has been reported that Na^+^ taken up into the mitochondrial matrix by SLC8B1 in exchange for Ca^2+^ derived from calcium precipitates upon matrix acidification controls hypoxic signaling via the mitochondrial respiratory chain [113]. Specifically, it was reported that the Na^+^ imported into the matrix reduces membrane fluidity through interaction with phospholipids. This in turn was shown to lead to the generation of reactive oxygen species (ROS) by altering certain elements of the electron transport chain, thereby promoting an adaptive short-time elevation of mitochondrial complex III-dependent ROS production during acute hypoxia. Indeed, inhibition of Na^+^ import via SLC8B1 was sufficient to prevent this pathway leading to adaptation to acute hypoxia [113].

While the mechanism of mitochondrial targeting of SLC8B1 has not yet been studied, the sequence of human SLC8B1 does not seem to contain a mitochondrial targeting sequence according to TargetP-2.0 predictions (likelihood 0.0016). This suggests that other internal targeting signals are present that direct the protein into the mitochondria, possibly similarly to SLC25, MPC and sideroflexin proteins.

Disease-releated mutations have not been reported for SLC8B1 thus far. However, residues responsible for the unique Li^+^-exchange capacity of SLC8B1 have been investigated in detail. Based on the determined structure of an archaeal NCX homologue, NCX_Mj, it was found that only 3 of the 12 ion-coordinating residues were shared with human SLC8B1 [114]. By mutating the 9 different residues to their human counterparts, it was possible to engineer a mutant of NCX_Mj that can also mediate Li^+^-dependent Ca^2+^ exchange [114]. In a later study, it was found that mutation of residue D471 to alanine can shift the selectivity toward Na^+^, while mutations at several positions can render SLC8B1 a Li^+^-selective exchanger [115]. All these positions cluster close to the Na^+^-binding sites shown by the X-ray structures of NCX_Mj [116,117] and the homologous H^+^/Ca^2+^ exchanger CAX_Af from the euryarchaeota *A. fulgidus* [118].

## 8. Additional Families with Members Proposed to be Localized in the IMM

### 8.1. The SLC9 Na^+^/H^+^ Exchanger Family

SLC9B2 (NHA2, Na^+^/H^+^ antiporter 2) might localize to the mitochondria [119,120], but this has been disputed [121]. Otherwise, SLC9B2 is more similar to prokaryotic Na^+^/H^+^ exchangers (NHEs) than to eukaryotic ones [121], and is the only human member of the Cation/Proton Antiporter 2 (CPA2) subfamily [122]. SLC9B2 does not seem to contain a MTS according to TargetP-2.0 predictions (likelihood 0).

### 8.2. The SLC1 Glutamate/Neutral Amino Acid Transporter Family

A splice variant of SLC1A5 (SLC1A5_var) was recently reported to have a mitochondrial localization and to function as the “long sought-after mitochondrial glutamine transporter” [123]. However, several inconsistencies urge us to treat this conclusion with caution. Firstly, the software the authors used for signal peptide detection (PrediSi) is designed to detect the signal peptides of proteins secreted through the Sec pathway [124], and is therefore unsuitable to detect a MTS. In contrast, methods that were specifically developed to detect MTS, such as TargetP-2.0, do not detect the presence of an MTS in SLC1A5_var (likelihood 0). Furthermore, the antibodies used by the authors to detect SLC1A5_var are claimed to “recognize the SLC1A5_var after peptide-N-glycosidase F (PNGase F) treatment” [123]. However, the only N-linked glycosylation sites on SLC1A5 are N163 and N212 [125], which are present in exon 1 that is in fact not present in the splice variant SLC1A5_var. Therefore, PNGase F treatment should not affect SLC1A5_var recognition. Due to the missing N-glycosylation sites, it is also likely, contrary to what the authors claim, that SLC1A5_var is not glycosylated. Finally, SLC1A5_var, in line with what is reported by the authors in their Figure 1A, is missing residues 2–203 of, but is otherwise identical to, canonical SLC1A5. According to sequence alignment of human SLC1 family members and the structure of a thermostable variant of the paralogous human SLC1A3 [126], this would mean that SLC1A5_var is missing the first 4 TMHs of the transporter protein, constituting most of the scaffold subdomain of the transporter. The importance of this region in transport is underlined by the fact that TMH3 forms part of the binding site for the allosteric SLC1A3 inhibitor UCPH_101_, and TMH1 has been proposed to interact extensively with the lipid bilayer, harboring a possible lipid-binding site [126]. Thus, these regions are likely to be important in SLC1A5 as well, and it is questionable whether SLC1A5_var could function as a transporter with such an N-terminal truncation. Therefore, given the above-mentioned issues with the antibody used and the detection of an MTS, it is somewhat doubtful whether SLC1A5_var is truly a mitochondrial glutamine transporter. As an alternative, glutamine may be converted to glutamate in the mitochondrial intermembrane space via the phosphate-dependent glutaminase GLS [127] that is thought to be attached to the outer surface of the inner mitochondrial membrane [128]. Glutamate that is generated may then cross the IMM by the mitochondrial glutamate carriers SLC25A22 or SLC25A12. On the other hand, should GLS face the intra-mitochondrial matrix, a mitochondrial glutamine carrier is required. The need for such a carrier has been reviewed in detail in [129]. However, to clarify this subject matter, a conclusive subcellular localization study of GLS in the mitochondrial inner membrane is still required, in order to reveal whether the enzyme is active on the intermembrane space or within the mitochondrial matrix.

## 9. Conclusions and Open Questions

The spectrum of primary and secondary active transporters in the mitochondrial inner membrane has greatly broadened in the past decade through the functional identification of mitochondrial pyruvate carriers, sideroflexins, and other mitochondrial transporters such as SLC8B1. This plurality of IMM transporters that show marked dissimilarity to SLC25 carriers, with no apparent common evolutionary history to the SLC25 family, hints that many other, as of yet unidentified secondary transporter families could exist in mitochondria. For the classical SLC25 mitochondrial carriers, their structure, targeting mechanism and transport properties are quite well-studied, but for the more recently identified proteins, structural information, and a general understanding of their transport mechanisms are still lacking. Interestingly, the sideroflexin (SLC56) protein family seems to share no significant sequence similarity to any protein with a known structure, and is therefore likely to possess a yet undescribed and novel structural fold. Further studies would be needed to clarify residues involved in substrate binding for sideroflexins. Another structurally enigmatic family of proteins are the LETM/SLC55 transporters, which likely function in a hexameric unit that can change conformation upon changes in pH, which can be a basis for an alternating-access mechanism [90]. Nevertheless, the transport mechanism of any single-helix membrane-spanning ion exchanger such as LETM1 has not been described yet. Despite the lack of information on many of these proteins, we aimed to summarize sequence elements involved in targeting and function of mitochondrial transporters, and have also suggested residues that could have a functional relevance based on sequence analysis of less well-characterized transporter families. The subsequent verification of the resulting hypotheses could greatly contribute to our understanding of their transport mechanisms. In this review, we have omitted the discussion of disease-causing mutations of SLC25 carriers, as these have been reported in detail in other articles of the present review series [49]. However, for non-SLC25 proteins, we have discussed the limited number of point mutations that are known to be linked to disease and involve single-residue changes. For these proteins, on the one hand, more information about their biological role and disease involvement would be desirable. On the other hand, for some transporters, e.g., pyruvate carriers of the SLC54 family, structural model building could help understand their transport mechanism and interpret certain disease-associated mutations. These endeavors can also potentially aid the generation of therapeutic modulators for future clinical applications.
